# The different expression of glycogen phosphorylases in renal clear cell renal carcinoma and chromophobe renal carcinoma

**DOI:** 10.1186/s12014-020-9270-0

**Published:** 2020-02-26

**Authors:** Yang Lu, Guangda Luo, Songbiao Zhu, Xu Wang, Yuling Chen, ZhouHuan Dong, Shiyu Wang, Jie Ma, Haiteng Deng, Di Wu, Jun Dong

**Affiliations:** 1 Department of Nephrology, Chinese PLA General Hospital, Chinese PLA Institute of Nephrology, State Key Laboratory of Kidney Diseases, National Clinical Research Center for Kidney Diseases, Beijing, China; 2 Department of Urology, Chinese PLA General Hospital, Fuxing Road 28, Beijing, 100853 China; 3 MOE Key Laboratory of Bioinformatics, School of Life Sciences, Tsinghua University, Beijing, China; 4 Chinese PLA No. 69241, Urumqi, China; 5 State Key Laboratory of Proteomics, Beijing Proteome Research Center, National Center for Protein Sciences, Beijing, China

**Keywords:** Clear cell renal carcinoma, Chromophobe carcinoma, Proteomics, Glycogen translation, Metabolism

## Abstract

**Background:**

The various pathogenesis between Clear cell renal carcinoma (CCRCC) and Chromophobe renal carcinoma (CHRCC) contributes to the different tumor growth rate and metastasis. In this study, we explored the distinct proteomic profiles between these two cancers and found different expression of glycogen phosphorylases in two cancers.

**Methods:**

We explored novel targets by proteomics. Five CCRCC cases and five CHRCC cases were selected for tandem mass tag-labeling liquid chromatography-mass spectroscopy (LC–MS). Gene ontology and KEGG pathway were applied for bioinformatic analysis. Glycogen phosphorylases were detected by Western blotting.

**Results:**

CHRCC were younger, more commonly female, and had larger tumors compared to those with CCRCC. 101 differentially expressed proteins (DEPs) in CCRCC and 235 DEPs in CHRCC were detected by LC–MS. It was found that disruption of metabolic pathways, epithelial cell differentiation, and cell response were the common characters for two tumor types. Activation of cell–cell adhesion and oxidation–reduction process stimulate CCRCC growth and epithelial cell differentiation and transferrin transport was involved in CHRCC growth, We also found that oxidative phosphorylation is activated in CHRCC and inhibited in CCRCC. More importantly, we found and confirmed that upregulation of glycogen phosphorylase liver type in CCRCC and glycogen phosphorylase brain type in CHRCC mediated differential glycogenolysis in the two tumor types, which could serve as potential therapeutic targets.

**Conclusion:**

We found different expression of glycogen phosphorylases in CCRCC and CHRCC by quantitative proteomics, which provides potential therapeutic targets in the future.

## Background

Kidney cancer is the twelfth most common carcinoma in the world. Clear cell renal carcinoma (CCRCC) is the most common subtype of renal carcinoma and accounts for approximately 80% of primary renal cancers. In contrast, chromophobe renal cell carcinoma (CHRCC) is a rare subtype of renal carcinoma and accounts for 5 to 10% of renal cancers. CCRCC and CHRCC originate from the renal proximal convoluted tubules and distal nephrons, respectively. The over-deposition of glycogen and lipids is a characteristic of CCRCC, while CHRCC has abundant cytoplasm with prominent cell borders (“vegetable cells”) and may not have classically described perinuclear halos [1]. The cells of CHRCC are larger than those of CCRCC and grow more quickly than CCRCC, but the metastatic rate is lower than in CCRCC. Moreover, patients with CHRCC have a more favorable outcome than those with CCRCC [2]. Different molecular mechanisms underlie the tumorigenesis and progression of these two cancers.

Many studies have explored the different mechanisms in CCRCC and CHRCC. The hepatocyte nuclear factor-1β gene is inactivated in CHRCC, but preserved in CCRCC and oncocytoma [3], and inactivation of protein polybromo-1 or BRCA1-associated protein-1 is less common in CHRCC than in CCRCC [4]. Vimentin-positive cells in CCRCC and CD9-positive cells in CHRCC can be used to distinguish CCRCC from CHRCC [5]. CHRCC cells derive ATP from oxidative phosphorylation [6], whereas CCRCC cells follow the Warberg effect [7]. However, there still lacks of effective therapeutic targets for two cancers. Therefore, we applied quantitative labeling liquid chromatography-mass spectroscopy (LC–MS) to explore novel therapeutic targets by building the proteomic landscapes profiles of the two cancers.

## Methods

### Patient information

We selected five CCRCC (mean age: 46 ± 5 years old, three males and two females) and five CHRCC (mean age: 47 ± 5 years old, three males and two females) patients who signed the consents in 2014 and 2015. These 10 patients had no metastasis. Tumors and adjacent normal tissues were collected for proteomic research. In avoid of long time storage, we extracted the proteins for all the samples and performed proteomics analysis once we got the last tissue. None of patients was treated with chemotherapy, radiation or other anti-tumor drugs prior to surgery. The assays were approved by PLA General Hospital ethics committee (Approval Number S2015-061-01).

### Protein digestion and liquid chromatography–tandem mass spectroscopic analysis

The tumors and adjacent normal tissues were ground with liquid nitrogen in a mortar and dissolved in fresh lysis buffer (8 M urea in PBS, pH 8–8.5, 1 mM PMSF, and 1 mM protease inhibitor cocktail). The digestion process was similar to that used in a previous study [8]. Briefly, protein content was determined using the BCA Protein Assay Kit (Thermo-Fisher Scientific, Rockford, IL, USA), in accordance with the manufacturer’s instructions. A 200 µg portion of protein from each sample was used for digestion. The proteins were incubated in a final concentration of 5 mM DTT at room temperature for 1 h and a final concentration of 10 mM IAA in the dark at room temperature for 30 min. Trypsin was added and the sample was incubated overnight at 37 °C. TFA was added to stop the reaction and a Sep-pack column was used to desalt the solution followed by mixing with tandem mass tag (TMT) for labeling and desalting again.

The TMT-labeled peptides were separated by 60 min of gradient elution at a flow rate of 0.250 µl/min using the EASY-nLCII™ integrated nano-high-performance liquid chromatography system (Thermo-Fisher Scientific). The analytical column was a fused silica capillary column (75 μm ID, 150 mm length; Upchurch, Oak Harbor, WA, USA) packed with C-18 resin. Mobile phase A consisted of 0.1% formic acid and mobile phase B consisted of 100% acetonitrile and 0.1% formic acid. The Q Executive mass spectrometer was operated in the data-dependent acquisition mode using Xcalibur 2.1.3 software and there was a single full-scan mass spectrum in the Orbitrap (400–1800 m/z, 60,000 resolution) followed by 10 data-dependent MS/MS scans at 27% normalized collision energy.

### Data analysis

The data analysis was similar to that described in a previous study [8]. Proteome Discoverer from UniProt (version PD1.4, Thermo-Fisher Scientific) was used to analyze the raw data with the human database. Precursor ion mass tolerances were set to 10 ppm, and the fragment ion mass tolerance was set to 20 mmu. Carbamidomethylation of Cys, TMT of lysine, and the peptide of the N terminal were set as the fixed modifications, while oxidation of Met was set as the variable modification. The peptide spectrum matched with the q value was < 1% as corrected peptide. The false discovery rate was set to 0.01 for protein identification. Quantification was carried out only for proteins with two or more unique peptide matches. Protein ratios were calculated as the median of all peptide hits belonging to a protein. Quantitative precision was expressed as protein ratio variability. Protein expression changes > 1.8-fold (tumors versus adjacent normal tissue) and p value < 0.05 (Student’s paired t-test) were considered to represent differentially expressed proteins (DEPs).

### Western blotting

The proteins were isolated using RIPA (pH 7.5 containing 50 mM Tris–HCl, 150 mM NaCl, 0.5% deoxycholate, 1% Nonidet P-40, 0.1% SDS, 1 mM phenylmethylsulfonyl fluoride, and a protease inhibitor cocktail). Glycogen phosphorylase liver type (PYGL), glycogen phosphorylase brain type (PYGB), and glycogen phosphorylase muscle type (PGYM) antibodies were purchased from the Proteintech group (Wuhan, China), and β-actin (Sigma-Aldrich, St. Louis, MO, USA) was used as the loading control. Approximately 30 µg of protein was loaded for 10% sodium dodecyl sulfate-polyacrylamide gel electrophoresis. The blots were developed with enhanced chemiluminescent reagent (Santa Cruz Biotechnologies, Santa Cruz, CA, USA), in accordance with the manufacturer’s instructions, and exposed to X-ray film. The protein bands were quantified using Quantity One software (Bio-Rad, Hercules, CA, USA).

### Immunohistochemistry assay

Paraffin-embedded sections (4-µm-thick) were stained with PYGL and PYGB (Proteintech group). A biotinylated secondary antibody and horseradish peroxidase (HRP)-streptavidin working buffer (Histostain Plus Kits, ZYMED, USA) were used for reaction and Vecta-stain DAB Kit (Vector Lab, USA) was used as the chromogen.

### Bioinformatic analysis

The DAVID website (http://david.abcc.nciferf.gov/) was used for the Gene Ontology and KEGG pathway analyses (p < 0.05 was set as a significant change). The String website (http://string-db.org/) was used for the protein–protein interaction (PPI) analysis.

### Statistical analysis

The Chi square analysis was applied to compare the sex distribution (%). The *t*-test was conducted for two-way comparisons. A p-value < 0.05 was considered significant.

## Results

### The clinical feature of CCRCC and CHRCC

In both CCRCC and CHRCC group, serum creatinine, serum uric acid, and random urine protein were in normal range. All the CCRCC patients and three CHRCC patients were in stage T1N0M0 phase and two CHRCC patients in stage T2N0M0. The tumor size in CHRCC group was greater than that in CCRCC (p < 0.05). All these information were presented in Table 1.

**Table 1:** The clinical information for CCRCC and CHRCC patients

Sample_ID	Gender	Age (years)	BMI	Tumor size (cm)	TNM stage	Fuhrman grade
CCRCC group						
CCRCC-1	Male	52	24.06	3	T1a	I
CCRCC-2	Male	46	26.23	1.5	T1a	I–II
CCRCC-3	Male	50	23.31	5.5	T1b	I–II
CCRCC-4	Female	39	21.89	6	T1b	II
CCRCC-5	Female	45	24.84	3	T1a	I–II
CHRCC group						
CHRCC-1	Female	46	28.05	20	T2	NA
CHRCC-2	Male	56	21.35	4	T1b	NA
CHRCC-3	Female	20	16.02	5	T1b	NA
CHRCC-4	Male	51	20.38	2.4	T1a	NA
CHRCC-5	Male	62	22.41	12	T2	NA

### The DEPs in CCRCC and CHRCC

A total of 159 DEPs in CCRCC (133 downregulated and 26 upregulated, Fold change > 1.8-fold versus control, p < 0.05) and 238 DEPs in CHRCC (162 downregulated and 76 upregulated, Fold change > 1.8-fold versus control, p < 0.05) were detected. Based on the expression trends, we divided the DEPs into four clusters. Cluster 1 included DEPs downregulated in the two renal cancers, which showed the common characters for two diseases; clusters 2 and 3 included DEPs mainly changed in CCRCC or CHRCC. Which showed specific pathogenetic mechanism for two cancers; and cluster 4 included DEPs with opposite changes of expression in the two cancers, which showed different mechanism between two cancers.

### Fatty acid, glutathione, glycine, serine and threonine metabolism, and oxidation–reduction were disturbed in the two tumor types

**Fig. 1:**
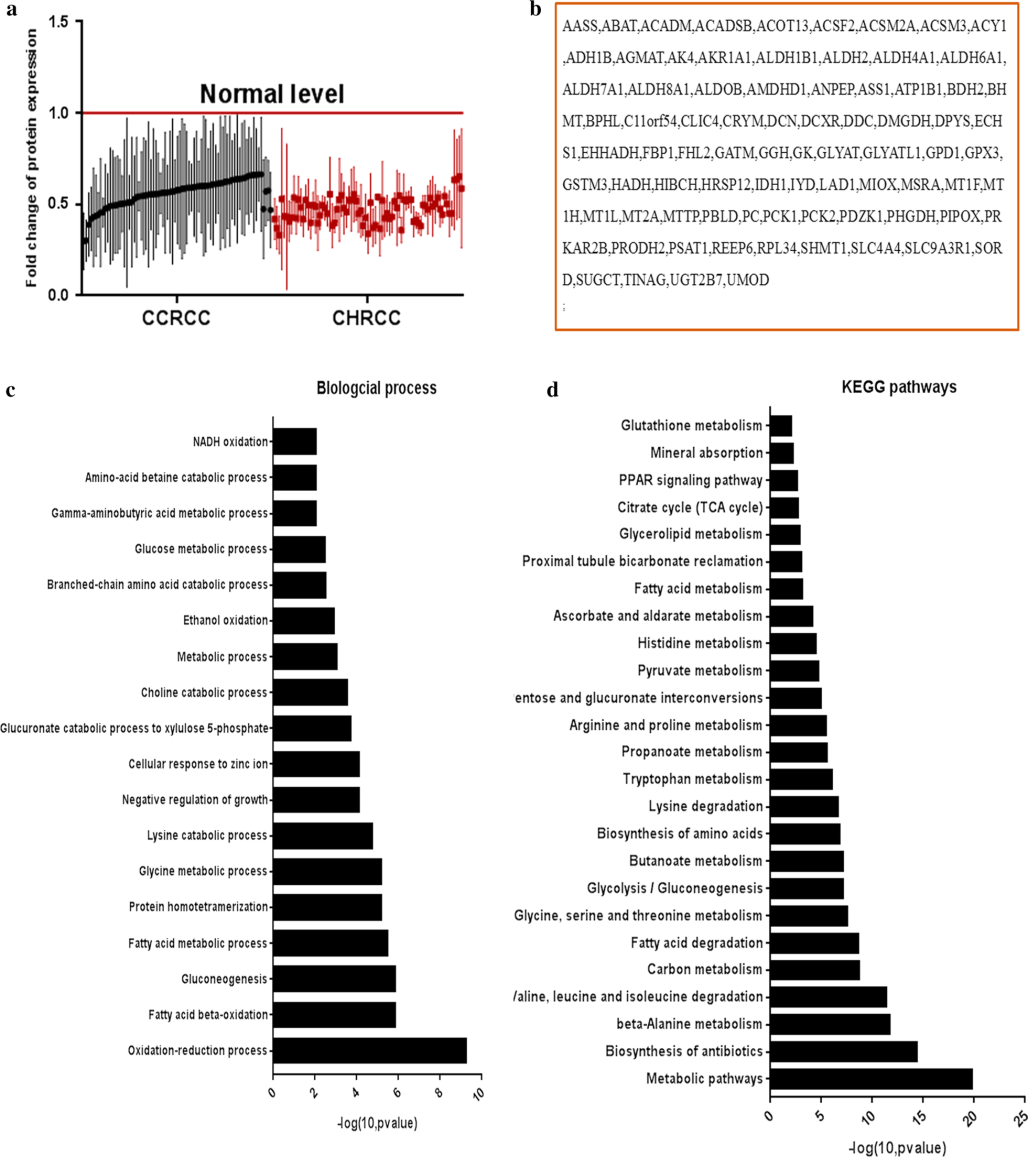
The common differentially expressed proteins (DEPs) in clear cell renal carcinoma (CCRCC) and chromophobe renal cell carcinoma (CHRCC). (Cluster 1) **a** Scatterplot results show that these common DEPs were downregulated (mean ± SD) compared to the controls in the two groups. **b** The list of DEPs. **c** The biological processes of the DEPs. **d** The KEGG pathways of the DEPs

The DEPs that changed in CCRCC and CHRCC showed the common mechanism of the two diseases. These common DEPs were mainly downregulated in the cluster analysis (Fig. 1a, b). We analyzed their biological functions and KEGG pathways (Fig. 1c, d). The disturbed metabolism and oxidation–reduction process were the main characters for the common DEPs in two tumors. Metabolism distribution included fatty acids metabolism (PRKAR2B, ACADSB, EHHADH, ACSM2A, HADH and ACSF2), glycolysis (ALDH7A1, AKR1A1, ALDH1B1, ALDOB, ALDH2, FBP1, ADH1B, PCK2 and PCK1), Glycine, serine and threonine metabolism (SHMT1, ALDH7A1, GATM, BHMT, PHGDH, DMGDH, PSAT1 and PIPOX), the tricarboxylic acid (TCA) cycle (IDH1, PCK2, PC, and PCK1), glutathione metabolism (GSTM3, GPX3, IDH1 and ANPEP) et al. These abnormal metabolic pathways were associated with oxidation–reduction (ALDH8A1, ALDH6A1, ACADM, SORD, AASS, PIPOX, IYD, MSRA, ALDH7A1, AKR1A1, GPX3, MIOX, PHGDH, DMGDH, PRODH2, ALDH4A1, CRYM and DCXR), which induced the tumor cells proliferation and metastases.

### Activation of cell–cell adhesion, oxidation–reduction process and glycolysis, and inhibition of the TCA cycle was associated with CCRCC progression

**Fig. 2:**
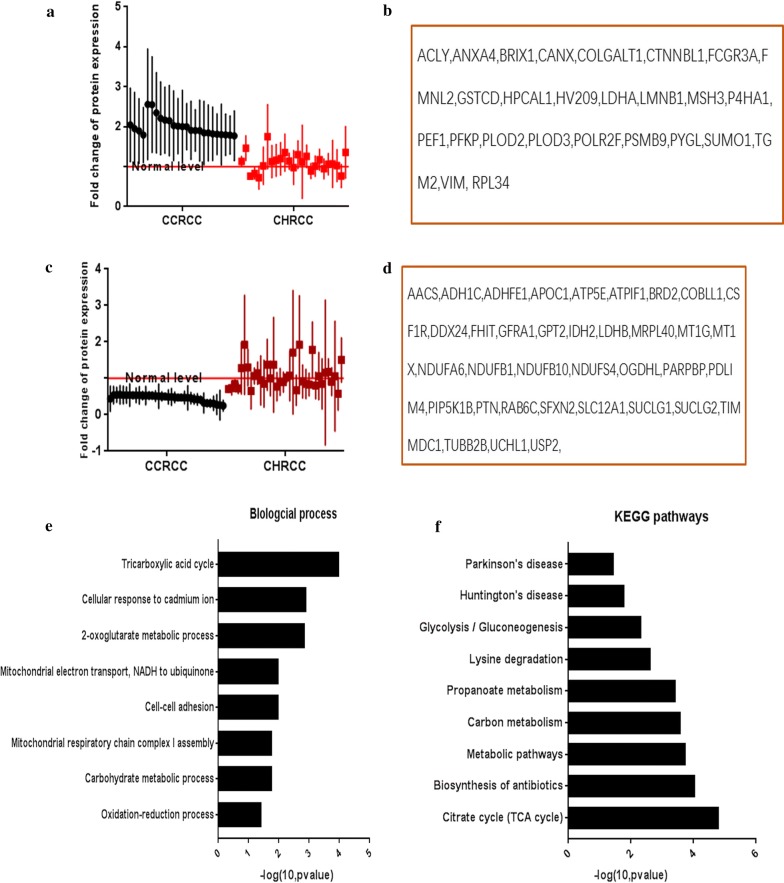
Independent differentially expressed proteins (DEPs) in clear cell renal carcinoma (CCRCC). **a** Scatterplot results of upregulated independent DEPs (mean ± SD) in CCRCC. **b** List of upregulated independent DEPs in CCRCC. **c** Scatterplot results of downregulated independent DEPs (mean ± SD) in CCRCC. **d** The list of downregulated independent DEPs in CCRCC. **e** The biological processes of independent DEPs in CCRCC. **f** The KEGG pathways of independent DEPs in CCRCC

The DEPs upregulated and downregulated independently in CCRCC are shown in Fig. 2a–f. These DEPs were closely related: TCA cycle (downregulation: SUCLG2, SUCLG1, OGDHL, and IDH2; upregulation: ACLY), activation of cell–cell adhesion (downregulation: COBLL1; upregulation: FMNL2, LDHA, RPL34, and PFKP), activation of oxidation–reduction processes (downregulation: LDHB and ADHFE1; upregulation: LDHA, PLOD2, P4HA1, and PLOD3), and activation of glycolysis/gluconeogenesis (downregulation: LDHB and ADH1C; upregulation: LDHA and PFKP). These results reveal the mechanism for energy supply to CCRCC and the regulation of cell growth.

### Epithelial cell differentiation, oxidation–reduction processes, glycolysis, and transferrin transport was involved in CHRCC growth

**Fig. 3:**
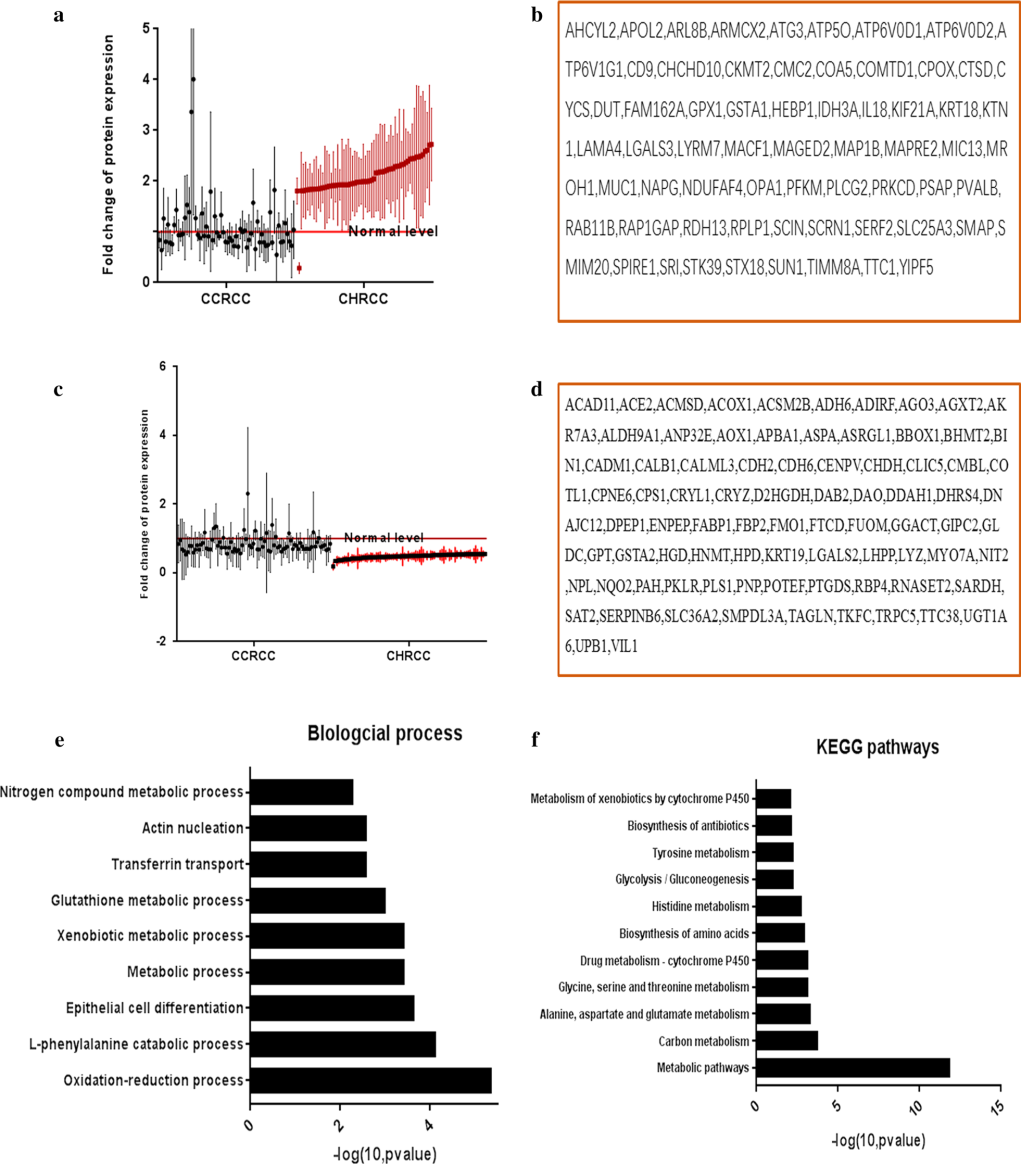
Independent differentially expressed proteins (DEPs) in chromophobe renal cell carcinoma (CHRCC). **a** Scatterplot results of upregulated independent DEPs (mean ± SD) in CHRCC. **b** List of upregulated independent DEPs in CHRCC. **c** Scatterplot results of downregulated independent DEPs (mean ± SD) in CHRCC. **d** List of downregulated independent DEPs in CHRCC. **e** The biological processes of independent DEPs in CHRCC. **f** The KEGG pathways of independent DEPs in CHRCC

The DEPs upregulated and downregulated independently in CHRCC were detected (Fig. 3a–f). These DEPs regulated biological functions, including oxidation–reduction processes, epithelial cell differentiation (upregulation: MUC1 and LGALS3; downregulation: GSTA1, GSTA2, TAGLN and VIL1), transferrin transport (upregulation: RAB11B, ATP6V1G1, ATP6V0D1, ATP6V0D2), and glycolysis/gluconeogenesis (downregulation: PKLR ADH6, ALDH9A1 and FBP2; upregulation: PFKM). These DEPs mediated energy metabolism and cell growth of CHRCC.

### Oxidative phosphorylation is activated in CHRCC and inhibited in CCRCC

**Fig. 4:**
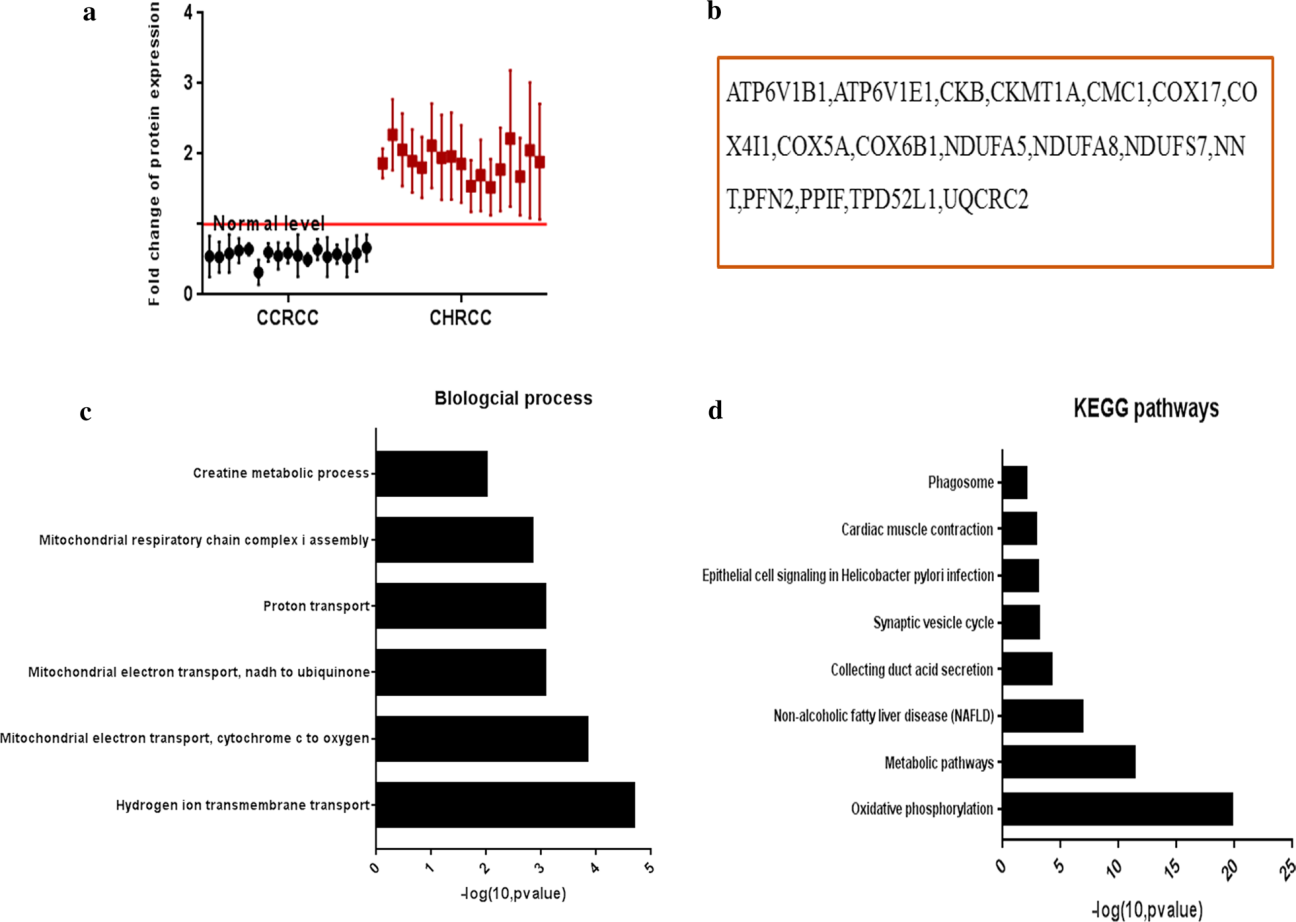
Differentially expressed proteins (DEPs) with opposite changes of expression in the two carcinomas. **a** Scatter plot results show that these DEPs (mean ± SD) are downregulated in clear cell renal carcinoma (CCRCC) but upregulated in chromophobe renal cell carcinoma (CHRCC). **b** The list of DEPs with opposite changes of expression. **c** Biological processes of DEPs with opposite changes of expression. **d** The KEGG pathways of the DEPs with opposite changes of expression

The DEPs that exhibited opposite changes of expression in the two carcinomas (DEPs downregulated in CCRCC and upregulated in CHRCC) were used to explore the different mechanisms underlying tumor development (Fig. 4a–e). Their biological functions included oxidative phosphorylation (UQCRC2, NDUFS7, NDUFA5, NDUFA8, ATP6V1E1, COX6B1, COX4I1, ATP6V1B1, COX5A and COX17), mitochondrial respiratory chain complex I assembly (NDUFS7, NDUFA5 and NDUFA8), and hydrogen ion transmembrane transport (NNT, COX6B1, COX4I1 and COX5A). Oxidative phosphorylation is the main energy supplying pathway and the activation of the opposite regulatory mechanisms in CCRCC and CHRCC reveals that energy for CHRCC depends mainly on oxidative phosphorylation, whereas energy for CCRCC depends on other energy supplying pathways, such as glycolysis.

### Glycogen phosphorylases were expressed differentially in CCRCC and CHRCC

**Fig. 5:**
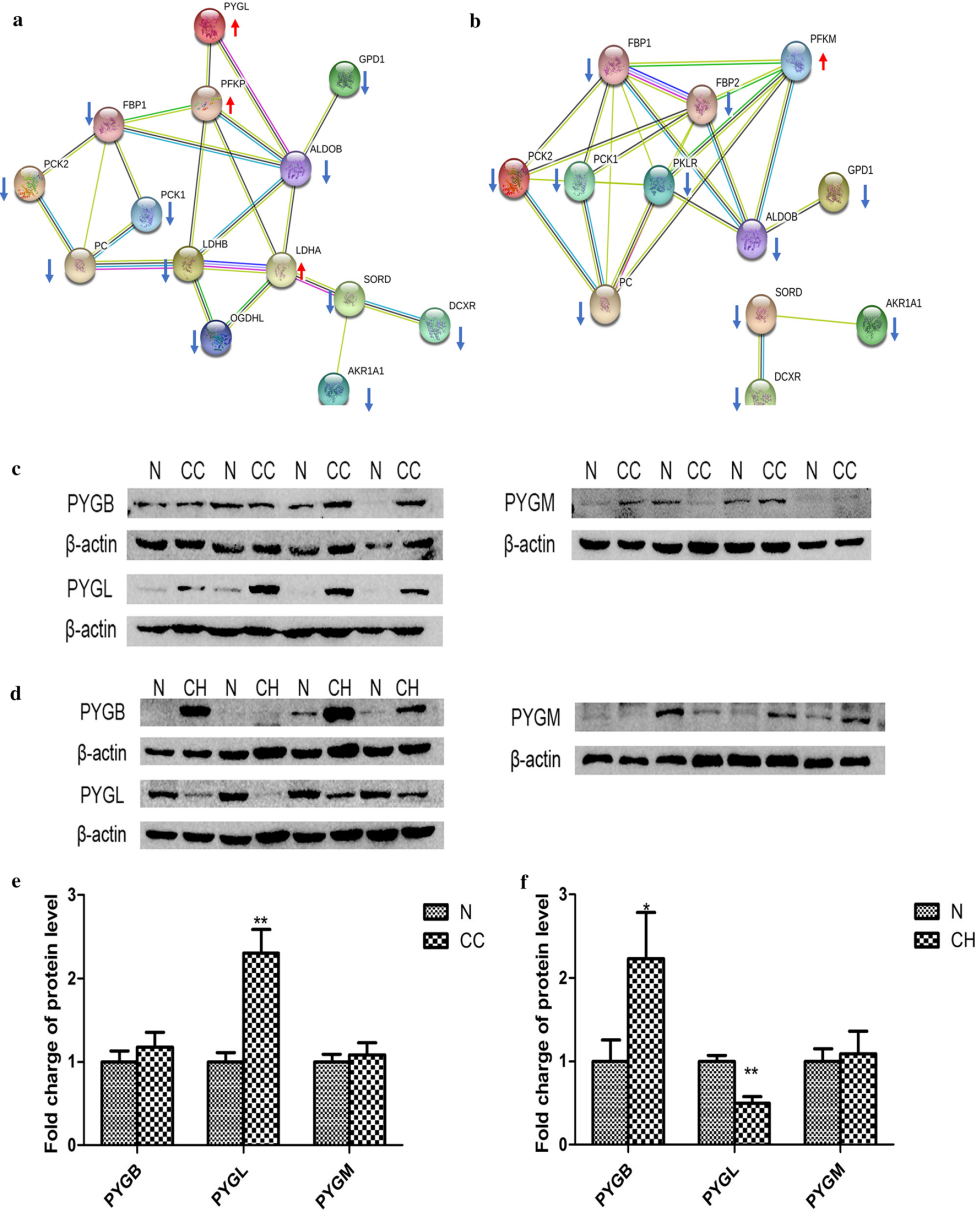
Different glycogen phosphorylases in clear cell renal carcinoma (CCRCC) and chromophobe renal cell carcinoma (CHRCC). **a** A protein–protein interaction (PPI) network for glucose metabolism in CCRCC. **b** PPI network for glucose metabolism in CHRCC. **c**, **d** Expression of glycogen phosphorylase liver type (PYGL), brain type (PYGB), and muscle type (PGYM) was detected in CCRCC and CHRCC by Western blotting. **e** Densitometry analysis shows that PYGL is upregulated in CCRCC. **f** The densitometry analysis shows that PYGL was downregulated and PYGB was upregulated in CHRCC. *p < 0.01, **p < 0.01 vs. control groups

**Fig. 6:**
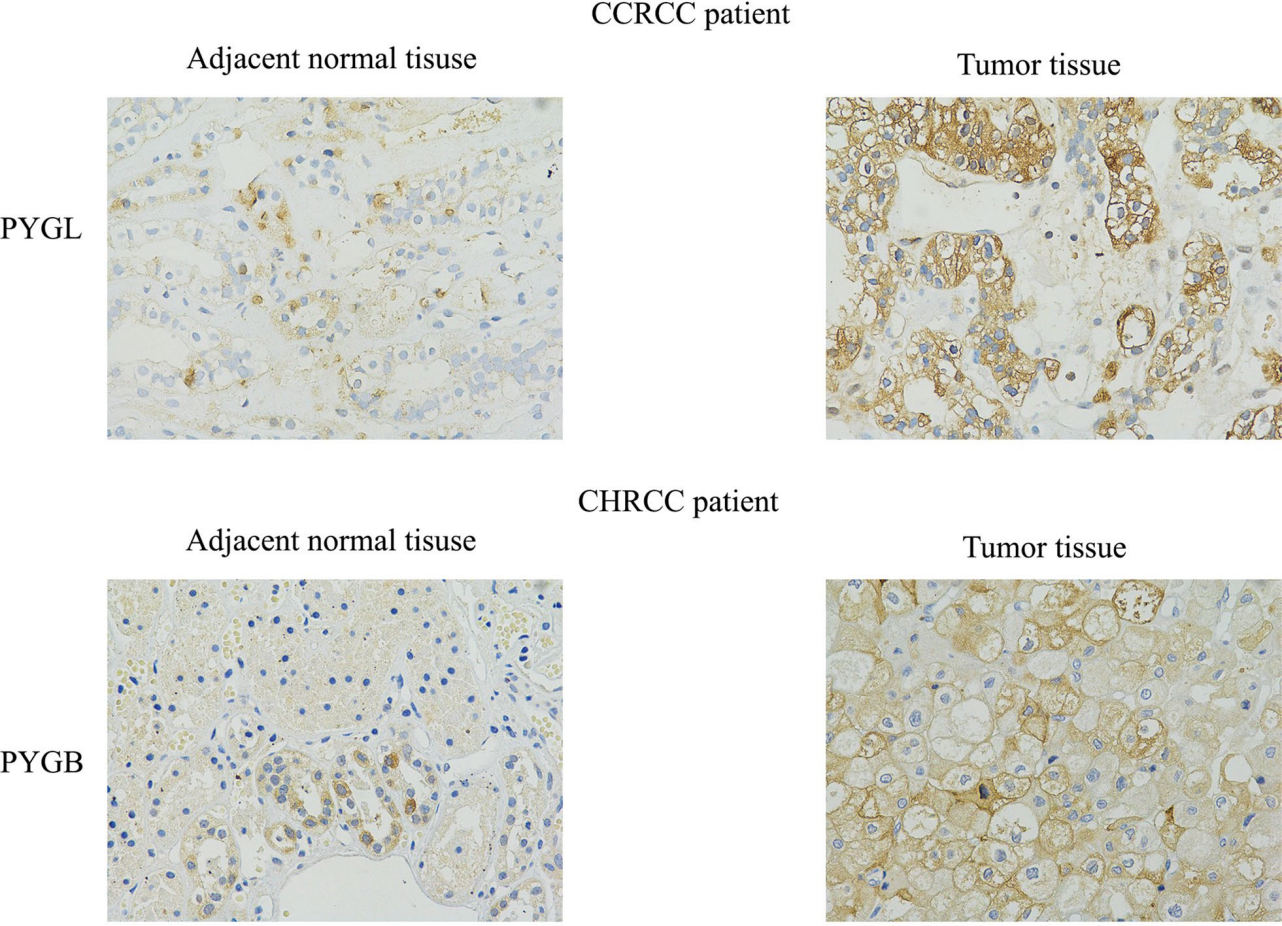
The expression of PYGL in CCRCC and PYGB in CHRCC were validated by immunohistochemistry assay (× 400). The expression of PYGL in CCRCC tumor tissue was higher than its adjacent normal tissue and the expression of PYGB in CHRCC tumor tissue was higher than its adjacent normal tissue (four CCRCC cases and three CHRCC cases)

We built a PPI network to explore the differences in glucose metabolism between CCRCC and CHRCC (Fig. 5a, b). The results show that PYGL was upregulated in CCRCC, but not in CHRCC. As a crucial glycogen phosphorylase, PYGL and two other glycogen phosphorylases, PYGB and PGYM, catalyze phosphorolysis of the α-1,4-glycosidic bond in glycogen to yield glucose 1-phosphate, which contributes to glycolysis and other energy metabolic functions. We detected these three glycogen phosphorylases in CCRCC and CHRCC to clarify the different mechanisms underlying glycogenolysis in the two tumor types Western blotting confirmed that PYGL was mainly upregulated in CCRCC and PYGL downregulation and PYGB upregulation were the features of CHRCC (Fig. 5c–f). We further applied immunohistochemistry assay to validate this results (Fig. 6). It was shown that PLGY were overexpression in CCRCC tumor tissue compared to its adjacent normal tissue and PYGB were overexpression in CHRCC tumor tissue compared to its adjacent normal tissue and PYGB. These results confirmed the differential expression of glycogen phosphorylases in CCRCC and CHRCC.

## Discussion

In this study, we first researched the different epidemiological characteristics of the two tumor types in China. We found that patients with CHRCC were younger, more commonly female, and had larger tumors compared to those with CCRCC, which was consistent with another Chinese renal carcinoma study [9]. However, more patients with CCRCC had metastases than patients with CHRCC. We deduced that these differences were dependent on a different molecular pathogenesis. Thus, we established proteomic profiles to explore the differential mechanism between the two tumor types.

The proteomic results suggested that reprogramming metabolism is the main characteristic of both tumor types. First, we found that many DEPs were common in the two tumor types, which disturbed the similar metabolism pathways to promote cancer cells proliferation in two tumor types. For example, glycolysis-associated enzymes such as phosphoenolpyruvate carboxykinase 1, 2 (PCK1, 2) and enzyme fructose-1,6-bisphosphatase 1 (FBP1), were downregulated in in two tumors, which resulted in glucose intake and kept tumor cells survive [10, 11]. Glutathione peroxidase 3 (GPX3), as a tumor suppressor, was suppressed in two tumors, could promote tumorigenesis [12, 13]. These proteins could be used as potential therapeutic targets for two cancers. However, we also found that aminopeptidase N (ANPEP)/CD13 was inhibited in two tumor types. ANPEP was thought as an important therapeutic target for cancers and inhibition of it could suppress tumor cells proliferation and migration [14, 15]. But ANPEP is not suitable as a therapeutic target in renal carcinoma.

The distinct mechanisms are crucial to explore the characters of two cancers. We found distinct mechanism in metabolism, TCA cycle, cell–cell adhesion and cell differentiation in two tumors. In CHRCC, downregulated peroxisomal acyl-coenzyme A oxidase 1 (ACOX1)-mediated fatty acid degradation and downregulated glutathione S-transferase alpha 1 and 2 (GSTA1 and -2)-mediated dysfunction of glutathione metabolism triggers oxidation and promotes CHRCC carcinogenesis [16–18]. While in CCRCC, upregulated-ATP citrate lyase (ACLY)-mediated fatty acid synthesis and lactate dehydrogenase A (LDHA)-mediated glycolysis provided ATP for CCRCC tumor cells growth. Moreover, the key regulatory enzymes in the isocitrate dehydrogenase (IDH) family, including IDH1, IDH2, and IDH3A, played differential in the two cancer types. In CCRCC, the downregulation of IDH1 and IDH2 inhibited TCA, while upregulated IDH3A may promote the progression of the TCA in CHRCC by inducing HIF-1 pathway to promote cell growth [19]. The distinct metabolism processes were associated with differential energy supplement in two tumors [6], which resulted in different tumor cells growth rate in two cancers.

Besides oxidative phosphorylation, cell–cell adhesion-related and cell differentiation-related proteins were shown to be involved in regulating the different statuses of cell growth and metastasis in the two tumor types. In CCRCC, formin-like protein 2 (FMNL2) and 60S ribosomal protein L34 (RPL34) were activated in CCRCC, which promoted cell growth and metastasis. While in CHRCC, mucin 1 (MUC1) and galectin-3 (LGALS3) were activated in CHRCC, which promote cell growth. FMNL2 regulates the invasiveness of cancer cells by driving β1-integrin internalization and RPL34 could promote cell growth and metastasis of oral squamous cell carcinoma [20, 21]. LGALS regulates renal epithelial growth and differentiation in CHRCC [22], and MUC1 interacts with LGALS on the cell surface to promote EGFR dimerization and activation in epithelial cancer cells [23]. These results proved that CCRCC and CHRCC had distinct mechanism for cell growth and metastasis.

Interestingly, we also found differential regulatory mechanisms underlying transition from glycogen to glucose in the two tumor types. Glycogen phosphorylases are crucial for catalyzing phosphorolysis of the α-1,4-glycosidic bond of glycogen to yield glucose 1-phosphate. We found that upregulation of PYGL in CCRCC could stimulate the glucose transition, whereas downregulation of PYGL and upregulation of PYGB mediated glycogenolysis in CHRCC. We deduced that CCRCC relied more on glycolysis because of increased glycogen deposition, whereas CHRCC relied more on glycolysis and the TCA cycle together. This finding suggests that glycogen phosphorylase inhibitors, particularly targeting PGYL and PYGB, may be useful for CCRCC and CHRCC therapy. PYGL inhibitors are already in development for treating type 2 diabetes and they are unlikely to be toxic to most cells because patients affected by Hers disease (an inherited glycogen storage disorder caused by deficiency of PYGL) [24]. The combination of a PYGL inhibitor and a HIF inhibitor could increase the efficiency of drugs to treat CCRCC. However, PYGB inhibitors are still in development.

In conclusion, we demonstrated that patients with CHRCC were younger, more commonly female, and had larger tumors than patients with CCRCC, which was dependent on their specific molecular pathogenesis. Based on the proteomic profiles, we found common and different mechanisms in the two tumor types. More importantly, we demonstrated that upregulation of PYGL in CCRCC and upregulation of PYGM in CHRCC may be targets for future therapy.

## Data Availability

All the data in the manuscript are available from the corresponding author and first author.
